# Brassicaceae Fungi and Chromista Diseases: Molecular Detection and Host–Plant Interaction

**DOI:** 10.3390/plants12051033

**Published:** 2023-02-24

**Authors:** Marwa Mourou, Maria Luisa Raimondo, Francesco Lops, Antonia Carlucci

**Affiliations:** Department of Agricultural Sciences, Food, Natural Resources and Engineering, University of Foggia, Via Napoli 25, 71122 Foggia, Italy; marialuisa.raimondo@unifg.it (M.L.R.); francesco.lops@unifg.it (F.L.)

**Keywords:** Brassicaceae diseases, molecular detection, brassica–host interaction

## Abstract

Brassicaceae plants cover a large number of species with great economic and nutritional importance around the world. The production of *Brassica* spp. is limited due to phytopathogenic fungal species causing enormous yield losses. In this scenario, precise and rapid detection and identification of plant-infecting fungi are essential to facilitate the effective management of diseases. DNA-based molecular methods have become popular methods for accurate plant disease diagnostics and have been used to detect Brassicaceae fungal pathogens. Polymerase chain reaction (PCR) assays including nested, multiplex, quantitative post, and isothermal amplification methods represent a powerful weapon for early detection of fungal pathogens and preventively counteract diseases on brassicas with the aim to drastically reduce the fungicides as inputs. It is noteworthy also that Brassicaceae plants can establish a wide variety of relationships with fungi, ranging from harmful interactions with pathogens to beneficial associations with endophytic fungi. Thus, understanding host and pathogen interaction in brassica crops prompts better disease management. The present review reports the main fungal diseases of Brassicaceae, molecular methods used for their detection, review studies on the interaction between fungi and brassicas plants, and the various mechanisms involved including the application of omics technologies.

## 1. Introduction

The Brassicaceae is a noteworthy plant family, previously named Cruciferae. It comprises significant crops, namely, the genus Brassica [1,2] and the essential model plant Arabidopsis thaliana [3]. Due to their distinctive essence, scent, and flavor, but mostly because of their widely acknowledged functional characteristics, the majority of Brassicaceae species are significant vegetables consumed around the world [4,5,6]. *Brassica rapa*, *Brassica napus*, and *Brassica oleracea* are the three most well-known species. However, *Brassica* vegetables are susceptible to several severe diseases that should be monitored to acquire desirable crop production [7]. Conventional techniques of control are predominately expensive with restricted efficiency and lead to ecological destruction; as an alternative, the typical approach is to use resistant genes of the *Brassica* crop hosts themselves [8]. Climate change, pathogen variability, and inadequate cultivation contribute to disease outbreaks, which hamper crop yield. Furthermore, many Brassicaceae cultivars are vulnerable to the same diseases and pests because of the close genetic relatedness of these species [9]. The considerable rise in Brassica cultivation worldwide has also contributed to novel disease outbreaks, some of which were not previously of interest [9]. Indeed, various pathogens can infect *Brassica* crops and cause economically significant yield losses in brassicas worldwide [10,11,12]. Phytopathogens such as plant fungi and insects represent a heavy challenge to Brassica plants; however, bacteria and viruses have a slight impact on production [13]. Among the fungal diseases, white rust, wirestem, sclerotinia blight, Alternaria leaf spot, blackleg, powdery mildew, fusarium wilt, downy mildew, and clubroot are considered the most impacting fungal brassica diseases. However, white rust, clubroot, downy mildew, and damping-off represent the most destructive chromista diseases of Brassicaceae [8]. For example, blackleg and light leaf spot caused huge damage to oilseed rape crop yields in Europe, followed by *Verticillium wilt*, Alternaria leaf spot, downy mildew clubroot, and powdery mildew [7]. Therefore, it is crucial to accurately and rapidly detect and identify the plant-infecting fungus in order to assist in the appropriate control of the disease [14]. In our context, molecular approaches have been applied to detect multiple pathogens simultaneously in Brassicaceae [15,16]. Additionally, advances in PCR-based techniques, such as real-time PCR, have enabled rapid and accurate detection and quantification and become more common in plant pathology through the use of the available portable PCR machines and simpler protocols [14]. DNA-based methods have become common tools for accurately diagnosing plant diseases. Advances in polymerase chain reaction (PCR) methods including nested, multiplex, quantitative, post, and isothermal amplification techniques, DNA- and RNA-based probe development, and next-generation sequencing represent new tools for molecular diagnostics of plant fungi. Thus, molecular diagnosis of crop diseases is becoming a field reality [17]. However, prior processes, including genomic DNA extraction, which effectively lyses fungal cells and recovers the DNA, as well as purification and quantification of extracted DNA, are necessary for these molecular approaches. For this purpose, various techniques for DNA isolation from plant-infecting fungi are available [18,19,20,21]. Additionally, modern methods for diagnosing fungal plant diseases employ different DNA extraction kits [22]. Due to the financial effect of phytopathogens on crops, it is interesting that interactions with pathogens have long been the ones that have received the greatest attention and study [23].

Although much is still unknown about the mechanisms of interaction between cruciferous and fungi, some cases have been reported where glucosinolates (GSLs) need to be directly degraded. The fungi may need to obstruct the synthesis and hydrolysis in the host before hydrolyzation into antifungal isothiocyanates [4,24]

In this review, we first describe the main fungal diseases of the Brassicaceae family and the diagnostic tools, mainly DNA-based molecular methods used for detection, and finally, we compile relevant research papers on the cruciferous–fungi interaction, pointing out the possible benefits, the known mechanisms involved, and the use of omics techniques to uncover the host–fungi interaction in Brassicaceae.

## 2. Most Impactful Fungal Brassica Diseases and Their Molecular Detection

**Figure 1:**
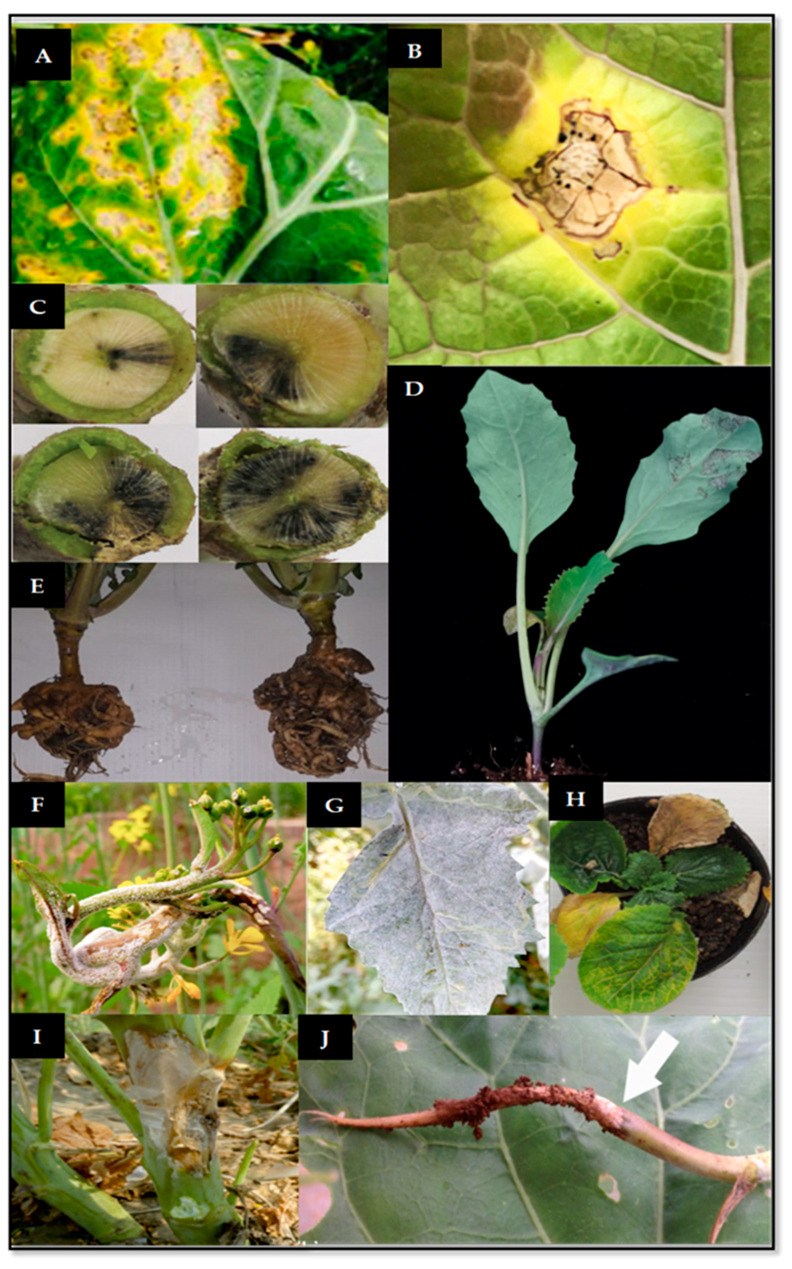
Representative symptoms of the main fungal diseases affecting Brassicaceae. (**A**) White leaf spot by *Neopseudocercosporella capsellae* [105]. (**B**) Leaf spot by *Alternaria brassicaea* [106]. (**C**) Blackleg by *Leptosphaeria maculans* [66]. (**D**) Downey mildew by *Perenospora parasitica* [11]. (**E**) Clubroot by *Plasmodiophora brassicae* [107]. (**F**) White rust by *Albugo candida* [108]. (**G**) Powdery mildew by *Erysiphe cruciferarum* [108]. (**H**) Fusarium yellows by *Fusarium oxysporum* f. sp. *conglutinans* [109]. (**I**) Sclerotinia stem rot by *Sclerotinia sclerotiorum* [108]. (**J**) Wirestem by *Rhizoctonia solani* [110].

The following section will give a general overview of the most impactful fungal brassica diseases (Figure 1) and their molecular identification through the different DNA-based detection methods (Table 1). A supplementary file is also available including nucleotide sequences of primers and probes cited in the present review (Supplementary File Table S1). 

### 2.1. Alternaria Leaf Spot

*Alternaria* spp. are considered to be the most virulent pathogen on all brassicaceous plants, with a wide host range including cabbage (*B. oleracea* var. *capitata*), Chinese cabbage (*B. campestris* var. *chinensis*), cauliflower *(B. oleracea* var. *botrytis*), broccoli (*B. oleracea* var. *italica*), canola (*B. napus*), mustard (*B. juncea*), and wild-grown plants [25]. Importantly, Alternaria diseases can result in significant yield loss [12,26] and represent one of the serious worldwide disease complexes, responsible, for instance, for reductions of up to 47% in Indian mustard *B. juncea*, and exceeding 70% in some *Brassica* species [12]. *Alternaria brassicicola* and *Alternaria brassicae* are the two species that most commonly cause Alternaria infections of crucifers, occasionally also *Alternaria alternata* [27]. However, a combination of three species, *A. brassicae*, *A. brassicicola*, and *A. japonica*, is the causal agent for the black spot, transmitted by the seed of numerous species belonging to genera *Brassica* and *Raphanus* [28]. Black spot disease, which has been observed in various crucifer hosts including *B. juncea*, *B. campestris*, and *B. rapa*, can be destructive to seed producers because it frequently causes premature pod shattering and shrunken seeds with decreased germination effectiveness [29,30]. Consequently, it causes a significant decline in crop production, including a decline in market value of cauliflower heads and oil quality in oilseed [15]. Due to the ability of both *Alternaria* species to persist in seed for long periods at various temperatures and humidity, a contaminated seed is the main source of primary inoculum for *A. brassicae* and *A. brassicicola* [15,31]. In fact, the conventional protocols often take about two weeks to detect them. In the literature, a few PCR-based methods have already been mentioned for the detection of *Alternaria* spp. on carrot [32], linseed [33], and cruciferous [34] seeds, as well as in host tissues [33] or food products [35]. The ITS region of nuclear rDNA [32,35] is the main genomic region targeted for PCR primer development. For instance, Iacomi-Vasilescu et al. [34] reported a sensitive and rapid PCR assay for the identification of *A. brassicicola* and *A. japonica* on cruciferous seeds [28].

Furthermore, various PCR-based methods have been conducted for detecting *A. brassicae* and *A. brassicicola* [36,37,38] separately, but not simultaneously. Therefore, Kiran et al. [15] developed a highly sensitive multiplex PCR protocol for fast and simultaneous detection. In detail, a series of novel primers, namely AbeABC1F and AbeABC1R, were designed based on ABC transporter (Atr1) gene associated with virulence. Results for sensitivity showed that both *A. brassicae* and *A. brassicicola* could be detected up to 100 pg of the DNA concentration. Therefore, this study could accurately detect both pathogens in a single PCR multiplex assay. Similarly, Guillemette et al. [28] also developed *A. brassicae*-specific markers from Atr gene regions and detected the fungus in infected seeds. Moreover, Aba28sF- and Aba28sR-specific primers for *A. brassicicola* were designed, targeting ABS 28 SSR region based on the study of Singh et al. [38] who demonstrated that ABS28 F and R primers distinguish the *A. brassicicola* from other closed pathogens. Consequently, multiplex PCR resulted in two specific 586 and 201 bp bands for *A. brassicae* and *A. brassicicola*, respectively. Additionally, Sharma et al. [39] developed a simple conventional polymerase chain reaction-based assay to detect and identify *A. brassicicola* through the use of specific primers designed based on ITS regions resulting in clear bands of ~600 bp using primers Acola-sens and Acola-reverse for *A. brassicicicola* isolates, with a detection limit of 100 pg [39].

**Table 1:** Summary of the main molecular techniques employed for detection of brassica fungal pathogens.

Molecular Technique	Detected Pathogen	Primer Name	Target Gene	Starting Material	Reference
Multiplex PCR	*Alternaria brassicae* *Alternaria brassicicola*	AbeABC1F and AbeABC1R Aba28sF and Aba28sR	ABC transporter (Atr1) gene ABS 28 based SSR marker	Pure DNA extracted from mycelium QIAamp^®^ DNA Mini Kit (Qiagen)	[15]
Conventional PCR	*Alternaria brassicicola*	Acola-sens and Acola-reverse primers	ITS region	Pure DNA extracted from fungal isolates and seed samples (CTAB Protocol)	[39]
Conventional PCR Real-Time PCR	*Alternaria brassicae*	ABCsens and ABCrev 115sens and 115rev	ABC transporter gene NRPS Gene (non-ribosomal peptide synthase)	DNA extracted from pure fungal cultures and seed macerates	[28]
Multiplex PCR	*Fusarium oxysporum* f. sp. *conglutinans* *Fusarium oxysporum*	Focs-1/Focs-2 W106R/F106S	Foc-specific fragments, whose length is 7 382 bp (Foc7382)	Pure DNA from plant (NuClean Plant Gen DNA Kit, Transgen Biotech Co., Ltd., Beijing, China) Pure DNA from fungal isolates following Lin et al. [40]	[17]
Real-time PCR Conventional PCR	*Fusarium oxysporum* f. sp. *conglutinans*	Cong_PG1_F., Cong_PG1_R., Cong_PG1_Probe PG1congF PG1congR	pg1 gene (endopolygalacturonase)	Pure DNA from from soil using FastDNA SPIN Kit (Q-BioGene, Montreal, QC, Canada) and from fungal isolates following Saitoh et al. [41]	[42]
Multiplex PCR	*Peronospora* *parasitica*	ITS4 and ITS5 (PpITS2F), (PpITS2R)	ITS region	Pure DNA extracted from fungal mycelium (CTAB Protocol)	[43]
Conventional PCR	*Leptosphaeria maculans* (*anamorphe: Phoma lingam*)	LmacF and LmacR	ITS region along with the 5.8S rRNA gene	Pure DNA extracted from imported canola seeds and pure cultures DNeasy Plant Mini Kit (Qiagen)	[44,45]
Conventional PCR	*Plasmodiophora brassicae*	TC1F and TC1R TC2F and TC2R	partial 18S ribosomal RNA (rRNA) gene 18S and ITS region	Pure DNA from mycelium, root, and soil (Roger and bendish [46] and Fast DNA spin Kit, Qbiogene Inc., Irvine, CA, USA)	[47]
Conventional PCR	*Albugo candida*	ACAN-1and ACAN-2 DC6 and LR-0	ITS1 region ITS region	Pure DNA extracted from symptomatic and asymptomatic plant tissue and surface-sterilized seed (CTAB protocol) Pure DNA extracted from leaf tissue; method described by Cenis et al. [48] and a modified protocol by Choi et al. [49]	[50] [51,52]
Real-Time PCR	*Pyrenopeziza brassicae*	OrSU677 and OrSU678 primers Probe: OrSU681	Cutinase gene Pbc1	Pure DNA extracted from symptomatic plant DNeasy Plant Minikit (Qiagen)	[53]
Conventional PCR	*Leptosphaeria maculans*	ITS1 and ITS4	ITS region	Pure DNA extracted from fungal cells DNeasy Plant Mini Kit (Qiagen)	[45]
Loop-mediated isothermal amplification (LAMP)	*Pythium ultimum*	SW-1 primer set (F3, B3, FIP, BIP, F-Loop)	target gene encoding a spore wall protein W-1	Pure DNA from pure cultures and infected plant tissues (CTAB method)	[54]
Quantitative PCR	*Rhizoctonia solani*	Specific primers: GMRS3-R GRSM4_M_ Probe: GRMP	ITS region	Pure DNA from mycelium, plant, and soil (Fast DNA Spin Kit, MP Biomedicals, Solon, OH, USA)	[55]
Conventional PCR	*Neopseudocercosporella capsellae*	ITS1 and ITS4	ITS region	Pure DNA extracted from mycelium (protocol adopted with some modification by Cenis [48])	[56]
Nested PCR Quantitative *PCR*	*Sclerotinia* *sclerotiorum*	ITS4/ITS5 and XJJ21/XJJ222 SSBZF and SSBZR Hydrolysis Probe: SSBZP	ITS region SS1G_00263	Pure DNA extracted from mycelium and petals (CTAB and microwave-based method)	[57,58]
Simplex and Multiplex PCR	*Sclerotinia minor* *Sclerotinia sclerotiorum*	SMLcc2 F SMLcc2 R SSaspr F SSaspr R	laccase 2 (Lcc2) aspartyl protease (Aspr)	Pure DNA from plants and fungal isolates following Sambrook and Russell [59]	[16]
Conventional PCR	*Erysiphe cruciferarum*	EryF and EryR	ITS region	Pure DNA from mycelium and infected plant tissue	[60]
Loop-mediated isothermal amplification (LAMP)	*Pyrenopeziza brassicae*	Pb_ITS_F3, Pb_ITS_B3, Pb_ITS_FIP, Pb_ITS_BIP, Pb_ITS_LoopF, Pb_ITS_LoopB, Pb_BTUB_F3, Pb_BTUB_B3, Pb_BTUB_FIP, Pb_BTUB_BIP, Pb_BTUB_LoopF, Pb_BTUB_LoopB	ITS region B-tubulin region	Pure DNA from Mycelium and Master Pure Yeast DNA Purification kit	[61]

Abbreviations: Foc, *Fusarium oxysporum* f. sp. *conglutinans*; ITS, internal transcribed spacer; B-tubulin, Beta Tubulin.

### 2.2. Blackleg Disease

*Leptosphaeria maculans* is the causal agent of blackleg disease of Brassicaceae and leads to enormous economic impact in the world [62,63]. The pathogen can survive on infected stems or other crop residues for many years in the shape of mycelia, pycnidia, and pseudothecia [64,65,66]. Chen et al. [45] identified *L. maculans* from imported canola seeds in China by (PCR) assay using a species-specific primer pair (LmacF/LmacR) [44]. In detail, DNA was extracted directly from the imported canola seeds using the DNeasy Plant Mini Kit (Qiagen China, Shanghai, China). Moreover, fungal species were also isolated from the imported seeds and the DNA was extracted from mycelia following the method previously described by Lee and Taylor [67] Additionally, a loop-mediated isothermal amplification (LAMP) was developed to detect *L. biglobosa “brassicae”* (Lbb). LAMP was optimized for temperature and time and tested for specificity and sensitivity using DNA extracted from Lbb, *L. biglobosa “canadensis”* (Lbc), *L. maculans* (Lm), and 10 other fungi. As a result, the optimum temperature and time were found to be 65 °C and 40 min, respectively. The LAMP primer set was specific and sensitive to Lbb, detecting as low as 132 fg of Lbb DNA per reaction. The LAMP assay was validated using DNA extracted from the mycelia and conidia of Lbb isolates, and its utility was demonstrated in DNA extracted from rapeseed leaves, stems, pods, and seeds [68].

Furthermore, isolates were identified as *L. maculans* or *L. biglobosa* by species-specific (PCR) assay conducted by Fernando et al. [69] The specific primers L. big F 5-ATCAGGGGATTGGTGTCAGCAGTTGA-3 along with the above-mentioned primers (LmacF/LmacR) were used in this research to identity each isolate as either *L. maculans “brassicae”* (Lmb) or *L. biglobosa “brassicae”* (Lbb). The PCR generates amplicons from *L. maculans “brassicae”*, which gave 331 bp, while *L. biglobosa “brassicae”* gave 444 bp [69].

### 2.3. Downy Mildew

Downy mildew caused by the oomycete *Hyaloperonospora parasitica* (syn. *Peronospora parasitica*) is prevalent in many cruciferous-crop-growing countries [70]. It is an obligate biotrophic parasite of Brassicaceae and allied families of Brassicales, including many relevant crops such as broccoli, cabbage, radish, and rape [71]. The disease is widespread globally wherever brassica crops are cultivated and is favored by a cold humid climate in spring or autumn. It has an impact on plants at all growing phases, but the most harmful impacts are mostly limited to young seedlings, leading to intense damage in plant nurseries and specific organs such as cauliflower curds. The lesions are usually yellow-green or yellow-brown on the leaf surface of the plant with white, powdery, mycelial growth on the underside of the leaf [72]. Other symptoms of downy mildew are large, irregularly shaped spots arising from multiple necrotic patches that coalesce to give the leaves a dry, yellow, or red color [21]. Concerning *Peronospora parasitica* detection, a research work carried out by Casimiro et al. [43] suggested the ITS multiplex PCR amplification, with primers ITS5, ITS4, and PpITS2F, as a reliable and simple method to identify *P. parasitica* in *B. oleracea*-infected tissues. In detail, the combination of ITS4 and ITS5 universal primers for amplifying the full-length ITS and a new specific internal forward primer (PpITS2F) for ITS2 generated a *P. parasitica*-specific amplicon appropriate for diagnostics. *B. oleracea* fungal pathogens and other downy mildew species are well-segregated, allowing for rapid, multiplex PCR amplification of full-length ITS of *P. parasitica*. The availability of a molecular marker based on the ITS2 spacer provides a PCR method that can be used as a trustworthy identification approach for *P. parasitica*, permitting early-stage detection of cruciferous downy mildew in seedlings and field plants and increasing seed strain screening [43].

### 2.4. Clubroot Disease

Clubroot Disease: Clubroot, caused by *Plasmodiophora brassicae*, is a worldwide soil-borne obligate parasite primarily affecting the Brassicaceae species [73,74]. *P. brassicae* is distinct from other plant pathogens, such as fungi or oomycetes [75,76]. It is potentially the most serious disease of cruciferous crops, especially cabbage and closely related crops. The disease has had an economic impact in many parts of the world. Clubroot can render infested fields unsuitable for the cultivation of Brassica plants due to the persistence of resting spores in the soil for up to 20 years. Representative symptoms of clubroot found on infected mustard, canola, and other Brassicaceae are root nodules with hard, white-looking bumps (“clubs”) that emerge firm and white in early infection, but gray and brown later when these galls mature and break down. All cruciferous species, including cruciferous plants and wild species such as *Arabidopsis thaliana*, are potential hosts of *P. brassicae.* The disease occurs in more than 60 countries and is most common in Brassica-intensive production areas such as Europe, Australia, and North America [21]. Indeed, all DNA-based methods have been exploited to test soil, water, or plant samples for clubroot. Several specific PCR-based diagnostic tests have been developed for *P. brassicae* and correlate well with disease severity. For instance, Buhariwalla et al. [74] developed PCR primers to specifically amplify the polymorphic DNA of *P. brassicae*. Ito et al. [75] developed semi-nested PCR for specific amplification of isopentyl transferase-like genes from *P. brassica* DNA [74,76]. These primers were then exploited to develop a protocol for the detection of *P. brassicae* in soil [75]. The PCR protocol was able to detect at least one resting spore per gram of artificially infected soil. However, for the detection, “dual” PCR was required, including another round with nested primers. Moreover, PCR assays have been conducted targeting the rDNA or ITS of *P. brassicae*. Chee et al. [77] used a PCR tool able to detect *P. brassicae* in infective roots of Chinese cabbage. However, soil detection has not been attempted. Faggian et al. [78] also conducted a nested PCR assay targeting the rDNA and ITS regions. The test was used with artificial inoculated soil and potting mix, infected soil, and with plant tissue [77]. Wallenhammar and Arwidsson [79] generated a nested PCR assay that enables the detection of *P. brassicae* in infested soils. Positive test results were detected with soils associated with a disease severity index higher than 21%. Additionally, Cao et al. [47] developed an rDNA one-step PCR assay that enabled proper detection of *P. brassicae* in naturally infested soil down to 11% of inoculum levels’ disease severity or 100 fg quantities of DNA. In addition, after three days post-inoculation, the assay permitted the detection of *P. brassicae* DNA on/in symptomless roots [47,79]. Interestingly, the study conducted by Cao et al. [47] developed a protocol that may confer an effective way for monitoring *P. brassicae* in plant and soil materials in a specific and fast manner. In detail, a simple, one-step PCR was developed to detect *P. brassica* in plant and soil samples. The primers TC1F and TC1R, based on a *P. brassicae* partial 18S ribosomal RNA (rRNA) gene sequence from GenBank, produced a 548 bp product in the optimized PCR. A second pair of primers, TC2F and TC2R, which amplified a fragment of the 18S and (ITS) 1 regions of the rDNA repeat, were also examined and produced a 519 bp band [47].

### 2.5. Fusarium Yellows

Fusarium yellows (FW disease, also called *Fusarium wilt*) is one of the most destructive diseases of cabbage and other crucifers. It causes enormous loss in crop production. Indeed, the most effective approach is to exploit disease-resistant/tolerant cultivars. *Brassica oleracea* is the most studied *Brassicaceae* crop for resistance to Fusarium yellows [80], as well as *Arabidopsis thaliana* [81,82]. Hence, a fast and accurate assay for earlier detection and identification of the phytopathogen is crucial for effective pathogen control [83,84]. Traditionally, methods for discriminating *Fusarium oxysporum* f. sp. *conglutinans* (Foc) from other formae species were based on morphology, and identification commonly relied on pathogenicity testing against the corresponding host [85]. While the results of the traditional techniques are reliable and precise, they need time and require the strongest knowledge of Fusarium taxonomy. In 2016, in a study conducted by Ling et al. [17] using comparative genomic analysis, a set of Foc-specific primers (Focs-1/Focs-2) was designed to amplify a specific sequence fragment of Foc, 436 bp in size. A multiplex PCR assay based on a designed primer set and a universal primer set for *Fusarium* species (W106R/F106S) was established to detect Foc strains. The multiplex PCR assay showed high specificity in the sensitive detection of Foc, and Foc was isolated from infected plant tissues as well as natural soil in the field. With adjusted PCR settings, the multiplex PCR shows high specificity in detecting Foc and is highly sensitive in detecting only 100 pg of purified Foc genomic DNA or 1000 spores in 1 g of twice-autoclaved soil. These species-specific primers are primarily designed based on housekeeping genes that can differentiate between *Fusarium* species but fail to detect distinctly different forms of Foc [86]. The LAMP assay performs one-step amplification and detection in less than 1 h, compared with 2–3 h in real-time PCR and conventional PCR, showing greater flexibility in pathogen detection. On the other hand, other methods of real-time PCR and loop-mediated amplification (LAMP) have also been used to detect Foc [17,40,87]. Nowadays, a wide range of molecular assays are being performed to accurately identify *F. oxysporum*, of which those based on the detection of pathogen DNA or RNA are the most prevalent [88].

However, expensive devices and reagents are required for real-time PCR detection [89]. Importantly, for the specific detection of *Fusarium oxysporum* f. sp. *conglutinans* (Foc), a primer set for PCR (PG1congF/PG1congR) and a primer–probe set (Cong_PG1_F/Cong_PG1_R/Cong_PG1_Probe) were designed for real-time PCR based on its endopolygalacturonase gene (pg1) sequence [42]. The study demonstrated that both primers sets specifically and quantitatively detected Foc that were generated based on Foc-specific single-nucleotide polymorphisms on pg1 sequence. PCR and real-time PCR using these primers and probes can distinguish Foc isolates from other pathogenic forms and non-pathogenic isolates of *F. oxysporum* [42]. Furthermore, a multiplex PCR approach was developed by Umesha et al. [90] for the rapid identification of the black rot disease *Xanthomonas campestris* pv. *campestris* and the Fusarium yellows, both of which first display symptoms that resemble one another. The primers, made specifically for *F. oxysporum* f. sp. *conglutinans*, were designed in the primer3 tool (http://www.bioinformatics.nl/cgi-bin/primer3plus/primer3plus.cgi/, accessed on 11 January 2023). The forward primer is 5′-CCGTAGCACTTAGTGCAATG-3′, while the reverse primer is 5′GCATTTCCATCGGTCACGATTG-3′. An hrpF primer specific to *X. campestris* pv. *campestris* and a primer specific to Foc were employed in a multiplex PCR. A 619 bp band unique to *X. campestris* pv. *campestris* and a 400 bp band specific to *F. oxysporum* f. sp. *conglutinans* were generated in the multiplex test [90]. Moreover, a study by Karlsson et al. [91] performed a Fusarium-specific PCR protocol for Fusarium community distinction in the field coupled with high-throughput sequencing. Karlsson et al. [91] ameliorated the protocol described by Edel-Hermann et al. [92] by substituting the first primer pair of the nested PCR, EF1/EF2, with a novel designed primer pair, Fa+7/Ra+6. The new primers notably evolved the phylogenetic diversity of *Fusarium* species through nested PCR and sequencing. In conclusion, the primer pairs Fa+7/Ra+6 and Fa/Ra accurately reflected *Fusarium* species composition in DNA mock communities analyzed with high-throughput sequencing. Thus, the method was successful in analyzing Fusarium communities in soil and plant material [92].

### 2.6. Sclerotinia Disease

*Sclerotinia sclerotiorum* is the causal organism of stem rot of Brassica and more than 500 hosts are widespread globally, including in Brazil, Canada, China, India, Europe, and Australia [93]. The infection manifests on a wide range of crops and is capable of infecting plant tissues above or below the soil surface at different developmental stages, causing seed yield losses of up to 80% as well as significant reductions in oil content and quality [21,94]. For the detection and identification of *Sclerotinia* species, several research studies were conducted. For instance, the simplex and multiplex PCR assays described by Abdelmajid et al. [16] are reliable, rapid, sensitive, specific, and cost-effective diagnostic methods for the majority of agriculture-associated species of *Sclerotinia* genus. In detail, for the aspartyl protease gene of *S. sclerotiorum*, the calmodulin gene of *S. trifoliorum*, the elongation factor-1 alpha gene of *S. homoeocarpa*, and the laccase 2 gene of *S. minor*, specific primers were designed. Multiplex PCR was used to amplify DNA fragments of various sizes, yielding 264 bp for *S. minor*, 218 bp for *S. homoeocarpa*, 171 bp for *S. sclerotiorum*, and 97 bp for *S. trifoliorum*. For the purpose of identifying *Sclerotinia* species in pure samples, these primer sets can be used singly or in a multiplex. However, Andrew and Kohn [95] conducted a single-nucleotide polymorphism (SNP) assay for the identification of *S. sclerotiorum*, *S. minor*, and *S. trifoliorum*. However efficient, SNP approaches may be inadequate for routine monitoring since they require experience and they are time-consuming and high-cost in comparison with multiplex PCR [16,95]. Additionally, Njambere et al. [96] designed a primer for the ITS region of *S. trifoliorum*, but after sequencing the PCR amplicons, some sequences were identified as *S. sclerotiorum*, so the primer was not specific. Similarly, Freeman et al. [97] reported a PCR assay for the detection of *S. sclerotiorum* fungal spores applicable to air samples. However, the primers specifically designed to detect *S. sclerotiorum* are identical to the sequences of *S. minor* and *S. trifoliorum* and can also detect these species. Therefore, accurate, sensitive, and cost-effective molecular tools to detect and discriminate between *S. homoeocarpa*, *S. minor*, *S. sclerotiorum*, and *S. trifoliorum* are still needed [96,97]. As reported by Yin et al. [98] the primer pair SSREV/SSFWD, designed by Freeman et al. [97], was sufficiently unspecific to discriminate *S. sclerotiorum* from *Botrytis* spp., even at a high annealing temperature during amplification. Furthermore, Yin et al. [98] developed a real-time PCR assay using the primer pair SsF/SsR to detect *S. sclerotiorum* on the petals of oilseed rape. Moreover, Roger et al. [99] designed a pair of primers, mtSSFor/mtSSRev, and a quantitative PCR to detect ascospores of *S. sclerotiorum*. These techniques are accurate and sensitive but require high-quality DNA to ascertain dependable results and are expensive and strenuous to perform on a vast scale [98,99]. A study by Quin et al. [57] established a quick and accurate method to detect the petal infection of oilseed rape (*Brassica napus*) by *Sclerotinia sclerotiorum* using a nested PCR technique. The universal fungal primer pair ITS4/ITS5 and the specific primer pair XJJ21/XJJ222 were used to perform the first and second rounds of PCR amplification, respectively. The specific primer pair XJJ21/XJJ222 was developed using the SNPs among nuclear rDNA ITS sequences of *Sclerotinia* spp., *Botrytis* spp., and other selected fungi. This assay can differentiate *Sclerotinia* spp. from other fungi, including *Botrytis cinerea*, a closely related and frequent cohabitant on oilseed rape petals, and can detect 50 fg genomic DNA, five ascospores of *S. sclerotiorum* in vitro, or 50 ascospores of *S. sclerotiorum* on one petal in approximately 6 h, even in the presence of a high background of oilseed rape DNA [57]. Additionally, in 2016, primers and a hydrolysis probe were designed by Zisman et al. [58] to amplify a 70 bp region of an *S. sclerotiorum*-specific gene, SS1G_00263. A hydrolysis-probe-based qPCR was developed that had a detection limit of 8.0 × 10^−4^ ng of *S. sclerotiorum* DNA and only amplified *S. sclerotiorum* DNA for a qPCR-based assay and enabled rapid and accurate estimation of infection severity [58].

### 2.7. Powdery Mildew

*Erysiphe cruciferarum* is the causal agent of powdery mildew disease, inducing significant crop loss impact worldwide [100]. The pathogen infects Brassica crops and was reported from different regions of the world including *B. juncea* in the USA, Australia [100,101], and several Asian and European countries [102]. In India, considerable yield loss reaching up to 70% occurred for *B. juncea* [103]. For molecular detection of *Erysiphe cruciferarum*, recent amplification of the ribosomal internal transcribed spacer (ITS) 1 was conducted by using oligonucleotides EryF and EryR using a purified DNA extracted from diseased plant tissues [60,104].

### 2.8. Damping-Off and Wirestem

*Pythium* spp. are important soil-borne pathogens that cause damping-off and root rot in a large number of plant species including members of the genus brassica, such as cabbage and cauliflower [54]. These soil-borne microorganisms can persist indefinitely in soil or plant debris. If seedlings are affected before emergence, it appears as poor germination. If the decay is after emergence, seedlings that fall over or die are said to “damp-off” [54]. In recent years, molecular detection methods based on the polymerase chain reaction (PCR) have been developed. Conventional and real-time PCR assays have been widely used for the specific diagnosis of more than 20 plant-pathogenic *Pythium* species [111]. For instance, Cullen et al. [112] used a conventional PCR to identify *P. ultimum* using specific primers targeting the internal transcribed spacer (ITS) region [112]. The ITS region of the rDNA has historically been used for the identification of *Pythium* spp. by sequencing due to a large number of sequences available in public databases as well as the level of interspecific variation observed [111]. Historically, in 1997, Kageyama et al. [113] conducted a study to sequence the rDNA of the ITS region of *Pythium ultimum* and Pythium group HS, designing species-specific primers for (PCR) assay to detect *P. ultimum* from infected seedlings. The sequence of the ITS region of *P. ultimum* shared the same identity as that of Pythium group HS, which ensures that HS group is an asexual strain of *P. ultimum*. Through this study, the primer pair K1+K3, designed on parts of the ITS, sequences amplified strains of *P. ultimum* and the HS group but not strains of 20 other *Pythium* species [113]. Similarly, for the rapid and accurate detection of *P. ultimum*, Shen et al. [54] developed a loop-mediated isothermal amplification (LAMP) method. A target gene encoding a spore wall protein was identified from the *P. ultimum* genome using a comparative genomics approach. A PCR assay was also performed using the outer primers F3 and B3 to compare sensitivity with the developed LAMP assay. Results revealed that the detection limit of LAMP was 1 ng μL−1 DNA, 1000 times more sensitive than PCR. Interestingly, the LAMP was successfully used to detect *P. ultimum* and assist mainly early diagnosis in the field; thus, appropriate phytosanitary measures could be launched [54].

*Rhizoctonia solani* is the causal agent of wirestem on Brassica crops, which can reduce marketable yield. It is a soil-borne fungal pathogen that lives in the soil and causes hypocotyl rot and damping-off and has been linked to significant losses in Europe. *R. solani* frequently causes hypocotyl symptoms such dark brown water-soaked lesions, wire-like appearance, hypocotyl girdling, and pinching off, which results in plant loss. Plants that are affected could have leaves that are colored orange to purple. Additionally, lesions associated with lateral root rot with pale brown to dark brown coloration, whereas taproot rot symptoms are typically dark brown lesions [21,114]. Plants with wirestem may be stunted and dark lesions of varying length and depth appear on the hypocotyl or the stem just above the soil line. *Rhizoctonia solani* inoculum can be evaluated using molecular detection methods [55,115]. A suite of real-time PCR assays shown to be specific for *R. solani* detection in naturally infected field soils was carried out through a study by Budge et al. [116]. A medium-scale technique for the extraction of DNA from soil has also been developed and primers and probe were successfully designed using sequences from b-tubulin gene [116]. In another research study performed by Wallon et al. [55], two specific and sensitive qPCR protocols were conducted for *R. solani* AG1-IB and *R. solani*. An amplicon of 90 bp was generated using specific primers (AG 1-IB-F3 and AG 1-IB-R) and a probe (AG 1-IB-P). A probe (GRMP) was designed for *R. solani* to be used with previously described GMRS3-R PCR primer [38] and a modified version of GMRS4 reverse primer. The AG 1-IB qPCR amplified all strains of *R. solani* AG 1-IB tested, and no PCR amplicon was generated for any non-target isolates [55].

### 2.9. White Rust

The oomycete parasite *Albugo candida* is a widespread pathogen of Brassicaceae crops causing white rust disease worldwide, including in Brazil, Canada, China, Germany, India, Japan, Korea, New Zealand, Pakistan, Palestine, Romania, Turkey, the United Kingdom, and the United States. It is actually considered as a member of the Oomycetes belonging to the family Albuginaceae included in the Kingdom Chromista. They are a group of organisms that are more closely related to golden-brown algae than they are to fungi. It is an obligate parasite that attacks at least 29 genera of crucifers, including major crops [50]. The yield loss is dependent on the disease severity, reaching up to 60% in *Brassica rapa* L. in Canada, 23–89.8% of *B. juncea* in India, and about 5–10% in Australia [108]. Molecular markers are sensitive, fast, and the most efficient tools for the identification and differentiation of *A. candida* isolates. Several research studies revealed that sequencing of the rDNA ITS region is a very robust marker to compare closely related species in the Oomycota phylum [117]. In the literature, the ITS and the Cytochrome c Oxidase subunit II (COX2) gene have been used by many authors for accurate species identification and detection of *A. candida* [118]. Kaur et al. [51] used 16 host differences for nine *A. candida* isolates and exploited ITS rDNA sequence to differentiate *A. candida* isolates into races. Petkowski et al. [119] identified on the basis of ITS, LSU, and COX2 of mtDNA three taxa in the *A. candida* complex and proved that the majority of Australian strains were the prevalent shape of *A. candida* [51,119]. Moreover, genetic diversity among 33 *A. candida* isolates has been inspected by Dev et al. [52] through DNA sequencing of the specific primers such as ITS region of rDNA, the forward primer DC6 and reverse primer LR0 and (COX2) region of mtDNA, and the forward primer (5′-GGCAAATGGGTTTTCAAGATCC-3′) and reverse primer (5′-CCATGATTAATACCACAAATTTCACTAG-3′) developed by Choi et al. [52,120]. A PCR protocol for the detection of ITS 1 of the ribosomal DNA (rDNA) of *Albugo candida* has been conducted in research performed by Amstrong [50]. Based on distinctions in the ITS 1 sequence between *A. candida* and *Lepidium oleraceum*, a primer pair was designed. The primers preferentially amplified the *A. candida* ITS 1 from samples in which the plant ITS 1 template predominated. *Albugo candida* ITS 1 DNA was detected in symptomatic tissue and several asymptomatic plants. The findings of the study imply that in the native host *L. oleraceum*, *A. candida* can occur as a latent asymptomatic infestation and can further be transmitted through seed. However, additional research is still needed to ascertain clear detection because the possibility of false negatives has not been rejected. Furthermore, similar PCR-based techniques have become widely used to detect *Albugo* spp. and other fungi within plant tissue [50]. Additionally, for selective PCR amplification of *A. candida*, primer DC6 (5-GAG-GGA-CTT-TTGGGT-AATCA-3) and LR-0 (reverse complementary to LR-0R) (50-GCT-TAA-GTT-CAGCGG-GT-30) were used successfully in a research study conducted by Kaur et al. [51,121,122] to detect *A. candida* infection [51].

### 2.10. White Leaf Spot

*Neopseudocercosporella capsellae* (white leaf spot disease) is an important disease on crucifers leading to enormous yield loss under cool and wet conditions [56]. It is an important disease across most oilseed-brassica-growing countries [121], destructive in regions such as France, the United Kingdom, Canada, the United States of America, and Australia [56,122,123]. *Neopseudocercosporella capsellae* has a wide host range, infecting diverse wild and cultivated crucifers, including vegetable brassicas and forage [56]. The fungal pathogen *Neopseudocercosporella capsellae* affects leaves along with stems and pods. When an infection is severe, lesions frequently merge to form irregularly shaped lesions that lead to premature defoliation. The creation of efficient control measures is hampered by a lack of knowledge about important aspects of the pathogen’s life cycle [21].

In the literature, detection and identity confirmation of *Neopseudocercosporella capsellae* involved sequencing of internal transcribed spacer region (ITS) of the rDNA operon, along with the comparison of sequences available for *N. capsellae* in GenBank. A portion of the nuclear rRNA operon was amplified with primers ITS1 and ITS4 using polymerase chain reaction (PCR). In particular, *N. capsellae* has a morphological plasticity that permits the switch from a yeast-like single-cell phase to the multi-cell hypha stage [124]. The identity of the structures was ascertained through PCR assay and sequencing of the ITS1 and ITS4 regions of blastospores taken from a yeast-like colony and hyphae from a pure mycelial colony [56].

## 3. Plant–Fungus Interaction in Brassicaceae

### 3.1. Beneficial Plant–Fungus Interaction in Brassicaceae

It is recognized that a huge part of fungal interactions with plants is mutualistic, resulting in advantageous effects for plant host and fungus [122,123]. The Brassicaceae family comprises various crops economically and scientifically interesting, such as *Arabidopsis thaliana*, and crops of the genus *Brassica*. Brassicaceae crops are known for the accumulation in their tissues of secondary metabolites known as glucosinolates (GSLs), primarily implicated in plant pathogen protection. Diverse interrelationships between plants and fungi comprise both mycorrhizal and endophytic fungi [125]. Mycorrhiza and endophytic fungi may as well grant plant resistance to abiotic and biotic disorders [4]. Additionally, they are distinguished by forming vesicles or arbuscules in the plant that establish a finely tuned relationship in which the plant receives mineral nutrients in exchange for carbohydrates and lipids [125,126]. Brassica crops are among the 30% of plant species that are not able to establish effective associations with mycorrhizal hosts. In fact, this evolutionary process of non-interaction may involve the loss of symbiotic genes and the existence of specific GSL pathways that correlate with the absence of mycorrhization ability [127,128]. However, Brassicaceae plants can develop beneficial relationships with endophytic fungi. This necessitates either a reduction in the host plant’s defensive responses or tolerance or the fungus must suppress the host plant’s defenses. The host plant can gain significant agricultural benefits once the Brassica–endophyte fungus symbiosis is established, including the promotion of plant growth, a rise in crop production, a greater stress tolerance, and management of phytopathogens and infections [129], while it supplies refuge and nutrients to the fungus. For instance, *Serendipita vermifera*, *A. alternata*, or *L. biglobosa* were mentioned previously to significantly raise root and stem biomass when they colonize the roots of plants such as cabbage and rapeseed [130]. Another example was described by Rozpadek et al. [131], who demonstrated that the fungus *Mucor* sp. regulated photosynthesis and carbon. This led to an increase in plant growth in *Arabidopsis* due to better CO_2_ assimilation and water use efficiency. For instance, given that mutant strains deficient in the ACCD gene failed to enhance root development, it appears to be important for root elongation caused by *Trichoderma asperellum* in canola roots [132]. Additionally, gibberellin (GA) production is directly related to the vegetative and reproductive growth of plants [133]. The endophyte *Neosartorya* sp. was able to secrete the gibberellic acids in Chinese cabbage roots, increasing plant biomass and length [134]. However, the mode of action beyond the relationship of endophytes with the host is not clearly revealed [4,135]. According to Yan et al. [129], the endophyte may have different mechanisms of action that contribute simultaneously to confer to the plant its advantages [136]. These mechanisms include facilitating nutrient access and improving nutrient uptake, raising photosynthetic rates, and controlling the activity of secondary metabolites. One of the primary beneficial functions of fungi endophytes is nutrient supply via nutrient uptake [4,126]. Brassicaceae plants, which are unable to form functional mycorrhizal symbiotic associations, are more susceptible to this benefit from fungus endophytes [128]. Endophytic fungi can raise the availability of nutrients for plants. In recent years, there has been a lot of research on the function of endophytes in enhancing plant tolerance to abiotic stresses [137]. Endophytic fungi can synthesize compounds that decrease plant stress, act directly on the stress agent to decrease it, or stimulate a specified reaction in the plant that raises stress tolerance. For instance, *S. indica* reduces gall aggregation by the pathogen by up to 60% and induces defensive reactions in the roots of Chinese cabbage, increasing flavonoid content and acting against the *Plasmodiophora brassicae* pathogen [138]. However, *S. indica* failed to effectively control clubroot in rapeseed plants, highlighting the plant–fungi specificity in the efficacy of biocontrol methods [4].

### 3.2. Host–Pathogen Interactions and Omics Technologies

Plant–pathogen interactions are complex and include a variety of interrelated molecular pathways. Determining which genes are expressed during infection in both the host and the pathogen, as well as how these genes influence other genes’ expression in the pathways, is essential for understanding these mechanisms [139]. We can better understand at a deeper level the host and pathogen interaction in brassica crops by using omics technologies, such as genomics, proteomics, transcriptomics, and metabolomics techniques. Recent advances in omics technology have enhanced the coverage of the plant transcriptome, proteome, and metabolome during pathogen attack and regulation of post-infection responses. Omics technologies also allow us to learn about the identification of new virulence factors of pathogens, their host targets, and disease prevention and diagnosis [140].

#### 3.2.1. Examples of the Application of Transcriptomics, Pangenomics, and Metabolomics in *Brassica crops*

Associative transcriptomics (AT) and bulked RNA sequencing (BSR-Seq) [24] are examples of integrative approaches that have made it possible to combine transcriptomic information associated with genome marker data to improve the ability to detect genomic loci, monitoring resistance or susceptibility in plants and virulence in phytopathogens. These techniques have simplified paths to identify potential genes that underlie these loci and to analyze the patterns of gene expression that occur when major Brassica crops are attacked by pathogens. For instance, three of the known R genes have been cloned and were discovered to encode leucine-rich repeats-receptor-like proteins (LRR-RLPs; Rlm2 and LepR3) as well as a wall-associated kinase-like (WAKL) protein (Rlm9) in *B. napus* resistance against blackleg [24]. *B. napus* and *L. maculans* have recently been studied using transcriptome sequencing [141]; both studies employed susceptible cultivars to measure pathogen and host gene expression. These investigations help to identify genes with potential pathogenicity (e.g., effectors) and resistance. Consequently, transcriptomic analysis can offer useful data on the host’s quantitative resistance to particular diseases [142]. Furthermore, *Brassica* species have a significant R gene repertoire, sometimes referred to as resistance gene analogs (RGAs), according to Pangenomics. Examples of RGAs include receptor-like protein kinases (RLKs), receptor-like proteins (RLPs), wall-associated kinases, and nucleotide-binding site leucine-rich repeats (NLRs), which mostly include the TIR-NBS-LRR (TNL) and CC-NBS-LRR (CNL) types [143,144]. Additionally, Abdel-farid et al. [13] investigated the metabolic interactions using 1H nuclear magnetic resonance spectroscopy among three cultivars of *B. rapa* and three phytopathogens, *L. maculans*, *A. niger*, and *F. oxysporum*. Infected *B. rapa* leaves were found to contain metabolites, such as phenylpropanoids, flavonoids, and glucosinolates, that are strongly associated with fungal infection. Subsequently, differences in metabolic responses to each type of fungal infection were evaluated. Plants infected with *F. oxysporum* produced more phenylpropanoids (sinapoyl malate, feruloyl malate, and 5-hydroxyferuloyl malate) and flavonoids (kaempferol and quercetin) than infections from both other fungi and were found to accumulate fumaric acid. In addition to variation by fungal species, host plant cultivar has a significant impact on metabolic alteration [13].

#### 3.2.2. Omics Technologies, Genome Editing, and Bioinformatics Methods

Using the various omics, supplemented with further fine-tuning of the bioinformatics techniques, will not only accelerate screening for favorable alleles in Brassica germplasm that promote resistance against the main phytopathogens but also identify and clone appropriate genes with high precision. Hence, deep molecular data will also enable us to reveal Brassica–fungus interactions; thus, the omics sources are frequently explored in phytopathology studies. Interestingly, through comparative and population genomic studies, it has been possible to understand the relationship of the phytopathogen to its evolution style and species variability [24,145,146]. The large amount of cruciferous genome resources in public databases assists in the analysis of the several complicated processes associated with cruciferous plant–pathogen interactions. Coupled with the integration of advanced bioinformatics methods and multi-omics datasets, in silico methods are robust tools that can answer a variety of research questions, from identifying candidate genes in both Brassica hosts and fungal pathogens to the evolutionary pathways of resistance mechanisms. Examples comprise the in silico identification of the blackleg resistance gene, LepR4, in the C genome of *B. oleracea* var. *capitata* [147] and the in silico exploration of 641 NBS-LRR-type disease resistance genes in *B. napus*, jointly highlighting the genomic variability of these genes in *B. napus* [148]. NGS-based BSA is one of the recent uses of omics in the screening of the Brassica–phytopathogen interaction. This approach includes a bulk/pool of DNA samples representing individuals with segregated phenotypes. Through the use of NGS, pools have been genotyped, either through RNA sequencing (BSR-Seq) or whole-genome resequencing, followed by the detection of QTLs through SNP calling (QTL-Seq). This integrated multi-omics and systems biology data can be used for breeding high-quality resistant Brassica plants in a more holistic and precise manner [24]. Although the CRISPR/Cas9 system is developing into a potent tool for plant genome editing, very few successful instances of genome editing in Brassica have been reported [149]. In one of these examples, a doubled-haploid genotype AG DH1012 (a broccoli-like Brassica) from the mapping population of *Brassica oleracea* var. *alboglabra* (A12DHd) × *B. oleracea* var. *italica* (Green Duke GDDH33) was found to have the GA4 gene knockout [150]. The CRISPR-Cas9 technique was efficiently used to modify the genome of the early-flowering characteristic in *B. rapa* [151]. The chitin-binding gene LmCBP1 in *L. maculans* was shown to be significantly expressed during infection with *B. napus*, and investigation with CRISPR-Cas9 revealed that it was also implicated in tolerance to H_2_O_2_ during the plant immune reply [145].

#### 3.2.3. Responses of *Brassica* spp. to Fungal Infection

Indeed, responses to infection of *Brassica* spp. by fungal pathogens include hypersensitive responses, such as necrostic cells close to arrested hyphae, phytoalexin, callose, and lignin secretion, aggregation of pectin in xylem vessels’ lumen, and induction of pathogenesis-related (PR) proteins including 1,3-b-glucanase and chitinase. In contrast to other host–fungal systems in which important complementary resistance and avirulence genes have been characterized [146], the genetics of the interaction between *Brassica* spp. and the fungal pathogen is very complicated, such as the interaction between *Brassica* spp. and *L. maculans.* This is due to several factors including a lack of standardization of homogeneous differential plant cultivars and virulence testing procedures among research groups working on this pathogen. Furthermore, control of inheritance of seedling (cotyledon) and adult (stem) resistance can be varied [152]. Several genes conferring cotyledon resistance are mapped in *B. napus* but none have been cloned as of yet; this is probably due to the complex nature of the cruciferous genome, which contains highly duplicated regions [153]. Nevertheless, as mentioned above, glucosinolates (GSLs) are defined as sulfur-containing substances reported commonly in *Brassica* genus. The rapport of resistant Brassica crops to various fungi and glucosinolate amounts is still ambiguous. Overall glucosinolate levels are not highly linked with pathogen resistance in diverse *Brassica* species. In fact, a negative connection between *Alternaria* disease severity and glucosinolate quantity was mentioned in *Brassica napus* [154]. However, with *S. sclerotiorum* infection, the level of indolic glucosinolate accumulation by the pathogen in *Brassica napus* was positively correlated with plant resistance [154]. These apparent discrepancies could reveal the lifestyles of the fungal pathogens [155] and their host ranges, [156], in addition to the amount of glucosinolates and their breakdown products secreted by the host plants [154].

## 4. Conclusions

The present review reports the main Brassica fungal diseases and their DNA-based molecular detection. Moreover, we reviewed studies discussing the brassica–fungus interaction and the mutual benefits and the potential application of the so-called omics technologies and bioinformatics to elucidate the mechanisms of interaction. Importantly, pathogen detection is a fundamental part of Brassicaceae disease control. In most cases, researchers seek the identification and detection of different genera in diseased plant tissues. The sequencing of the ITS region has been commonly applied for primer design [157,158]. The feasibility of multiplexing assays is mainly associated with the successful hybridization of primers to targeted DNA, while amplicons’ size differences could be discriminated using gel electrophoresis [111]. As the fields of microbiology progress through multi-omics and next-generation sequencing, a better understanding of the host and pathogen interaction in brassica crops will be feasible. Nevertheless, a more detailed study on Brassica–fungal interactions, including the different mechanisms involved, is still needed.

## Data Availability

Not applicable.

## Supplementary Materials

The following supporting information can be downloaded at: https://www.mdpi.com/2223-7747/12/5/1033/s1. Supplementary Table S1: Primers and probes sequences of different fungal pathogens of Brassicaceae.
