# Correction: Mourou et al. Brassicaceae Fungi and Chromista Diseases: Molecular Detection and Host–Plant Interaction. *Plants* 2023, *12*, 1033

**DOI:** 10.3390/plants12173109

**Published:** 2023-08-30

**Authors:** Marwa Mourou, Maria Luisa Raimondo, Francesco Lops, Antonia Carlucci

**Affiliations:** Department of Agricultural Sciences, Food, Natural Resources and Engineering, University of Foggia, Via Napoli 25, 71122 Foggia, Italy

The authors wish to make the following corrections to this paper [1]:

In the article titled: “Brassicaceae Fungal Diseases: Molecular Detection and Host–Plant Interaction”. It would be better to modify the title and the keyword and also add a few sentences in the text where some pathogens are wrongly defined as fungi, but they belong to the Chromista Kingdom and/or Oomycota Phylum. There was only a problem with taxonomic aspects, and the changes regard only the systematics. Therefore, we would like to change the Title:

“Brassicaceae Fungi and Chromista Diseases: Molecular Detection and Host–Plant Interaction”. In the keywords: Instead of “Brassicaceae fungal diseases”, we will keep only the words “Brassicaceae diseases”.

A few sentences were adjusted in the text in the following part:

1. Introduction: However, white rust, clubroot, downy mildew, and damping-off represent the most destructive Chromista diseases of Brassicaceae [8].

2.3. Downy Mildew: It is an obligate biotrophic parasite of Brassicaceae and allied families of Brassicales, including many relevant crops such as broccoli, cabbage, radish, and rape [71].

2.4. Clubroot Disease: Clubroot, caused by *Plasmodiophora brassicae*, is a worldwide soil-borne obligate parasite primarily affecting the Brassicaceae species [73,74]. *P. brassicae* is distinct from other plant pathogens, such as fungi or oomycetes [75,76].

2.9. White Rust: It is actually considered as a member of the Oomycetes belonging to the family Albuginaceae included in the Kingdom Chromista. They are a group of organisms that are more closely related to golden-brown algae than they are to fungi.

The authors apologize for any inconvenience caused and state that the scientific conclusions are unaffected. The original publication has also been updated.
