# Supplementary material for: Brassicaceae Fungi and Chromista Diseases: Molecular Detection and Host–Plant Interaction
Source: Plants (Basel). 2023 Feb 24;12(5):1033. doi: 10.3390/plants12051033 (PMC10005080; doi:10.3390/plants12051033)
Supplement: Supplementary file 1 [file plants-12-01033-s001.zip › plants-2199565-supplementary.pdf]

Table S1: Primers and probes sequences of different fungal pathogens of Brassicaceae

Target	Primers and Probes	Nucleotide sequence
Alternaria brassicicola	Acola-sens	5′ -GCA GCA TCT GCT GTT GGG G-3′
	Acola-reverse	5′ -CAA GGT CAG CAT CCA TAA AGC C-3′
Leptosphaeria maculans	LmacF	5′-CTTGCCCACCAATTGGATCCCCTA-3′
	LmacR	5′-GCAAAATGTGCTGCGCTCCAGG-3′
Hyaloperonospora parasitica	PpITS2F	5′-AAGCGTGACGATACTAATTTG-3′
	PpITS2R	5′-TGAAGTGCGGCCGAAGCTT-3′.
Plasmodiophora brassicae	TC1F	5′-GTGGTCGAACTTCATTAAATTTGGGCTCTT-3′
	TC1R	5′-TTCACCTACGGAACGTATATGTGCATGTGA-3′
Fusarium oxysporum f. sp. conglutinans	Cong_PG1_F	5′-TGATCTTTCCAAGCTCAAGGATG-3′
	Cong_PG1_R	5′-GCTAATGACGATAGGATCAAAGTCG-3′
	Cong_PG1_Probe	5′-ACTTTTGCCACTACTGCTGA-3′
Sclerotinia sclerotiorum	SSaspr F	5′-TCAAACGCCAAAGCTGTATG-3′
	SSaspr R	5′-TCCTAGATCGACTCTCCTCCTTT-3′
Erysiphe cruciferarum	EryF	5′-TACAGAGTGCGAGGCTCAGTCG-3′
	EryR	5′-GGTCAACCTGTGATCCATGTGACTGG-3′
Rhizoctonia solani	GMRS3-R	5′AGT GTT ATG CTT GGT TCC ACT-3′
	GRSM4M	5′CGG TTC RTC TGC ATT TAC CTT-3′
	Probe: GRMP	FAM-CRG CGT GAT AAR TTA TCT ATC GC
Albugo candida	DC6	5′-GAG-GGA-CTT-TTGGGT-AATCA- 3
	LR-0	5′-GCT-TAA-GTT-CAGCGG-GT-3
Neopseudocercosporella capsellae	ITS1	5′TTT CCG TAG GTG AAC CTG C3′
	ITS4	5′TCCTCC GCT TAT TGA TAT GC3′
